# Induction of endogenous γ-globin gene expression with decoy oligonucleotide targeting Oct-1 transcription factor consensus sequence

**DOI:** 10.1186/1756-8722-2-15

**Published:** 2009-03-27

**Authors:** Xiaoxin S Xu, Xin Hong, Gan Wang

**Affiliations:** 1 Institute of Environmental Health Sciences, Wayne State University, 2727 Second Avenue, Detroit, MI 48201, USA

## Abstract

Human β-globin disorders are relatively common genetic diseases cause by mutations in the β-globin gene. Increasing the expression of the *γ*-globin gene has great benefits in reducing complications associated with these diseases. The Oct-1 transcription factor is involved in the transcriptional regulation of the γ-globin gene. The human *γ*-globin genes (both A*γ *and G*γ*-globin genes) carry three Oct-1 transcription factor consensus sequences within their promoter regions. We have studied the possibility of inducing *γ*-globin gene expression using decoy oligonucleotides that target the Oct-1 transcription factor consensus sequence. A double-stranded 22 bp decoy oligonucleotide containing the Oct-1 consensus sequence was synthesized. The results obtained from our *in vitro *binding assay revealed a strong competitive binding of the decoy oligonucleotide for the Oct-1 transcription factor. When K562 human erythroleukemia cells were treated with the Oct-1 decoy oligonucleotide, significant increases in the level of the *γ*-globin mRNA were observed. The results of our western blots further demonstrated significant increases of the fetal hemoglobin (HbF, α2γ2) in the Oct-1 decoy oligonucleotide-treated K562 cells. The results of our immunoprecipitation (IP) studies revealed that the treatment of K562 cells with the Oct-1 decoy oligonucleotide significantly reduced the level of the endogenous *γ*-globin gene promoter region DNA co-precipitated with the Oct-1 transcription factor. These results suggest that the decoy oligonucleotide designed for the Oct-1 transcription factor consensus sequence could induce expression of the endogenous *γ*-globin gene through competitive binding of the Oct-1 transcription factor, resulting in activation of the *γ*-globin genes. Therefore, disrupting the bindings of the Oct-1 transcriptional factors with the decoy oligonucleotide provides a novel approach for inducing expression of the *γ*-globin genes. It also provides an innovative strategy for the treatment of many disease conditions, including sickle cell anemia and β-thalassemia.

## Introduction

Human β-hemoglobin disorders, such as sickle cell anemia and β-thalassemia, are relatively common genetic diseases that affect millions of people worldwide. The diseases cause severe clinical symptoms including heart disease, stroke, kidney failure, infection, and other complications. It is well documented that these diseases are caused by mutations in the β-globin gene: a T-to-A mutation at the sixth amino acid codons of the β-globin gene causes sickle cell anemia and various deletions that occur at the β-globin gene lead to β-thalassemia [1]. When *γ*-globin genes are highly expressed, the presence of high levels of fetal hemoglobin (HbF, α2γ2) in erythrocytes (~20–30%) can compensate for the defective β-globin product and significantly reduce disease symptoms [1]. Therefore, increased expression of the *γ*-globin genes has important clinical relevance in the treatment of β-globin disorders. However, the *γ*-globin genes are developmentally regulated and normally expressed at high levels only during the fetal stage of human development [2-5]. In adults, the β-globin gene is predominantly expressed and the adult hemoglobin (HbA, α2β2) consists of over 98% of total hemoglobin whereas the *γ*-globin genes are expressed at very low levels and the HbF consists of less than 1% of the total hemoglobin. Several strategies have been developed to induce expression of *γ*-globin genes for the treatment of sickle cell anemia and β-thalassemia [6-9]. However, new strategies still need to be developed so that more effective treatments can be provided for these patients.

Oct-1 is a member of the POU family of transcription factors that specifically interacts with the octamer motif ATGCAAAT, a regulatory element that is important for tissue- and cell-specific transcription as well as for the transcription of a number of housekeeping genes [10]. Studies reveal that the promoter region of each of the human *γ*-globin genes carries three Oct-1 transcription factor consensus sequences, which are located at the -280, -220, and -175 regions, respectively [1]. Clinical studies reveal that mutations occurring at the -175 Oct-1 consensus sequence of the *γ*-globin gene lead to elevated levels of *γ*-globin transcription and increased levels of HbF in individuals with a hereditary persistence of fetal hemoglobin (HPFH) condition [11-13]. In our previous studies, mutations generated at the -280 Oct-1 consensus sequence of the *γ*-globin genes also resulted in increased transcription of the genes [14]. All of these observations suggest that the Oct-1 transcription factor negatively regulates transcription of the *γ*-globin genes, and therefore, disrupting the binding of the Oct-1 transcription factor at these consensus sequences may lead to increased expressions of the *γ*-globin genes.

Decoy oligonucleotides provide an attractive approach to manipulating transcription factors and regulating the expression of the desired target genes [15-19]. Theoretically, when a decoy oligonucleotide containing the consensus sequence of a specific transcription factor is introduced into the cells, the presence of high levels of the decoy oligonucleotide will compete with the endogenous gene targets for binding to the transcription factor, which will lead to the removal of the transcription factor from the endogenous gene targets and cause alteration in transcription of the target genes. Most published studies utilize the decoy oligonucleotides to down regulate transcription of target genes [20-29]. If decoy oligonucleotides can also be used to up regulate expression of the desired target genes, it will significantly extend its potential applications. Induced transcription of target genes with decoy oligonucleotides will also have important clinical implications in the treatments of many disease conditions.

We have studied the possibility of using decoy oligonucleotides targeting an Oct-1 transcription factor consensus sequence to induce expression of the endogenous *γ*-globin gene. Using a double-stranded decoy oligonucleotide containing the Oct-1 consensus sequence, the results obtained from our *in vitro *protein binding study revealed a strong competitive binding of the decoy oligonucleotide for the Oct-1 transcription factor. When K562 human erythroleukemia cells were treated with the decoy oligonucleotide, significant increases in the level of *γ*-globin mRNA and HbF protein were detected. The results obtained from our immunoprecipitation (IP) study further demonstrated that the treatment of the K562 cells with the decoy oligonucleotide caused a significant decrease in the binding of the Oct-1 transcription factor to the endogenous *γ*-globin gene promoter region DNA. All of these results suggest that the decoy oligonucleotide designed to target the Oct-1 transcription factor consensus sequence can effectively induce expression of the endogenous *γ*-globin genes by competing with the Oct-1 consensus sequences of the endogenous *γ*-globin gene for the Oct-1 binding, resulting in activation of the endogenous *γ*-globin gene and increased accumulation of the HbF protein.

## Materials and methods

### Cell line and oligonucleotides

The K562 Human erythroleukemia cells were purchased from the American Type Culture Collection (ATCC, Rockville, MD) and maintained in RPMI 1640 medium supplemented with 10% heat-inactivated fetal bovine serum.

All the oligonucleotides used in this study were synthesized either by the Keck Oligo Synthesis Laboratory at the Yale University School of Medicine or by Integrated DNA Technologies, Inc (Coralville, IA) and are listed in Table 1. A pair of phosphorothioate-modified complementary 24 mer oligonucleotides containing the Oct-1 consensus sequence was synthesized and annealed to form a 24 bp double-stranded DNA fragment, which was designated as the Oct-1 decoy oligonucleotide. A pair of phosphorothioate-modified complementary 24 mer oligonucleotides containing a scrambled sequence was also synthesized and annealed to form a 24 bp double-stranded DNA fragment, which was designated as the scrambled oligonucleotide.

**Table 1:** Oligonucleotides used in the study.

**Name of oligonucleotide**	**Sequences of the oligonucleotide**
1. Gel shifting Oct-1 oligos.	

Oct-1 binding probe (sense strand)	^5'^-CTGATACGATTTGCATACTGACGT-^3'^
Oct-1 binding probe (anti-sense strand)	^3'^-GACTATGCTAAACGTATGACTGCA-^5'^

2. Decoy oligonucleotides	

Oct-1 Decoy oligo (sense strand)	^5'^-TGTCGAATGCAAATCACTAGAA-^3'^
Oct-1 Decoy oligo (anti-sense strand)	^3'^-ACAGCTTACGTTTAGTGATCTT-^5'^

3. Control oligonucleotides.	

Scrambled oligo (sense strand)	^5'^-AGTCGTCACGTAAGTCGAGCAC-^3'^
Scrambled oligo (anti-sense strand)	^3'^-TCAGCAGTGCATTCAGCTCGTG-^5'^

4. Real time PCR primers.	

Forward primer	^5'^-TGGTGACCGTTTTGGCAATC-^3'^
Reverse primer	^5'^-GAAAGCTCTGCATCATGGGC-^3'^

### *In vitro *Oct-1 binding assay

A pair of complementary 24-mer oligonucleotides containing the Oct-1 consensus with a sequence of ^5'^ACGTCAGTATGCAAATCGTATCAG^3' ^was synthesized. The complementary oligonucleotides were annealed to form a 24 bp Oct-1 consensus DNA fragment. The DNA fragment was radioactively labeled with [γ-^32^P]-ATP by T4 polynucleotide kinase. The radioactively labeled Oct-1 consensus DNA fragment (1 × 10^-11 ^mole or ~20,000 cpm radioactively labeled Oct-1 DNA fragment) was incubated with a HeLa nuclear extract (5 μg) in a volume of 20 μl containing 1× Protein Binding buffer (20 mM HEPES, pH 7.9, 100 mM KCl, 0.2 mM EDTA, 0.5 mM DTT, and 20% glycerol) at room temperature for one hour. To reduce unspecific binding, denatured salmon sperm DNA (4 *μ*g) was also supplemented in each binding reaction. The unlabeled 24 bp scrambled or Oct-1 decoy oligonucleotide was added into some reactions for competitive binding of the Oct-1 transcription factor. The reactants were analyzed by polyacrylamide gel electrophoresis using a 6% gel and visualized by autoradiography.

### The Oct-1 decoy oligonucleotide treatment and preparation of total RNA

The K562 cells were seeded at a density of 4 × 10^4 ^cells/ml and treated with the Oct-1 decoy oligonucleotide at various concentrations by adding the 24 bp Oct-1 decoy oligonucleotide directly into the cell growth medium. As controls, some K562 cells were treated with either 10 *μ*M scrambled oligonucleotide or 75 *μ*M hemin, an effective γ-globin gene inducer, in parallel experiments. At various time points, the cells were harvested and total RNA was isolated using an RNeasy mini kit (Qiagen, Santa Clarita, CA). Total RNA was also isolated from the untreated K562 cells at the same time points in parallel experiments.

### Real time PCR assay

A two-step real time PCR assay was performed to measure the level of the *γ*-globin mRNA. The reverse transcription (RT) reaction was carried out in 2 μg of total RNA using the Taqman Reverse Transcription Master Mix (Applied Biosystems, Foster City, CA). A primer optimization step was then tested to determine the optimal primer concentrations. Once the optimal primer concentrations were determined, the cDNA sample (10 ng) was used for a quantitative PCR reaction to determine the value of cycle threshold (*C*_*t*_) of the *γ*-globin gene from each RNA sample using a Sybr Green Master Mix with ABI 7500 Fast Real Time PCR System (Applied Biosystems). The *Ct *value of the *GADPH *gene was also determined for each RNA sample. The real time PCR data was then analyzed to determine the levels of the *γ*-globin mRNA in each RNA sample. The level of the *γ*-globin mRNA in the untreated K562 cells of each time point was counted as 100% and the level of the *γ*-globin mRNA in the treated K562 cells of the same time point was then calculated as a percentage in comparison to that of the untreated K562 cells. The *GAPDH *gene was used as an internal control for normalization. Relative expression of the *γ*-globin mRNA was expressed as 2^-*ΔΔCt *^where *ΔC*_*t *_was calculated by subtracting the average normalization gene *C*_*t *_(*GAPDH*) from the average target gene (*γ*-globin gene) *C*_*t *_value in the same cell line and the *ΔΔC*_*t *_was obtained by subtracting the *ΔC*_*t *_of the untreated cells from the *ΔC*_*t *_of the treated cells.

### Western blot hybridization assay

The mouse anti-human fetal hemoglobin (HbF) monoclonal antibody (MHFH00) was purchased from Caltag Laboratories (Burlingame, CA). Both the untreated and the treated K562 cells were harvested and lysed in RIPA cell lysis buffer (1 × PBS, 1% NP-40 (v/v), 0.5% Deoxycholic acid (w/v), 0.1% SDS (w/v)). The cell lysates (30 *μ*g total protein) were analyzed by PAGE using a 10% gel. The proteins were transferred to a PVDF membrane and the level of HbF protein was determined by western blots using the mouse anti-human HbF antibody. The same membrane was then stripped in a stripping solution (62.5 mM Tris, pH6.8, 2% SDS (v/v), 0.7% 2-mercaptoethanol (v/v)) and hybridized with an actin antibody (Oncogene Research Products, San Diego, CA) to determine the level of actin in each sample. The level of the HbF was calculated as a level relative to that of the actin in each sample to minimize the experimental variations.

### Immunoprecipitation (IP) assay

The K562 cells were seeded at a density of 6 × 10^4 ^cells/ml. The cells were treated with either the 24 bp Oct-1 decoy oligonucleotide (2 *μ*M and 10 *μ*M) or the 24 bp scrambled oligonucleotide (10 *μ*M) for four days and then harvested. Approximately 6 × 10^6 ^cells were harvested for each study in the experiment. As a control, the same number of cells was also harvested from the untreated K562 cells. The cells were fixed in 1% formaldehyde for 20 minutes at room temperature and washed in 1 × PBS three times. The cells were resuspended in the SDS cell lysis buffer at a density of 1 × 10^6 ^cells/200 *μ*l and incubated on ice for 10 minutes. The cells were then sonicated (four cycles of 10 second sonications with a 30 second pulse) to lyse the cells. The cell lysates were centrifuged at 4°C for 10 minutes at 14,000 rpm to remove any insoluble cell debris and the supernatants were used in the IP study. The cell lysates (100 *μ*l) were diluted with 900 *μ*l of ChIP dilution buffer (0.01% SDS, 1.1% Triton X-100, 1.2 mM EDTA, 16.7 mM Tris-HCl, pH8.1, 167 mM NaCl) and then incubated with 20 *μ*l of Oct-1 antibody (Santa Cruz Biotechnology, Inc., Santa Cruz, CA) at 4°C overnight. Protein A-conjugated agarose beads (40 *μ*l beads) (Sigma Inc., St. Louise, MO) were then added and the reactants were incubated at 4°C for two hours. The beads were collected from the reactants by centrifugation and washed three times with low salt washing buffer (0.1%SDS, 1.1% Triton X-100, 2 mM EDTA, 20 mM Tris-HCl, pH8.1, 150 mM NaCl) and three times with high salt washing buffer (0.1% SDS, 1% Triton X-100, 0.5 mM EDTA, 20 mM Tris-HCl, pH8.1, 500 mM NaCl) to remove any unspecific binding proteins. Half of the beads were analyzed by western blot to determine the level of the Oct-1 protein precipitated with the beads in each reaction. The rest of the beads were resuspended in 100 *μ*l of 50 mM NaCl and incubated at 65°C for 5 hours to reverse the DNA-protein crosslinks. The proteins were removed by phenol chloroform extraction and the DNA was recovered by ethanol precipitation. The DNA was dissolved in 10 *μ*l of TE buffer and a quantitative PCR assay was performed to determine the amount of the *γ*-globin gene promoter region DNA contained in each reaction using a pair of primers that bind to the *γ*-globin gene at the positions of -350 to -330 and +50 to +30 respectively with the ABI 7500 Fast Real Time PCR System. The *Ct *value was determined for each reaction. The amount of *γ*-globin gene promoter DNA precipitated from the untreated K562 cells by the IP was calculated as 100% and the amount of *γ*-globin gene promoter DNA precipitated from the treated K562 cells by the IP was calculated as a percentage in comparison to that of the untreated K562 cells.

### Statistical analysis

Results are expressed as the mean + S.D. Statistically significant differences were determined using a one-factor analysis of variance with *p *< 0.01. The quantification of the γ-globin mRNA in these studies was obtained from at least three independent experiments.

## Results

### The Oct-1 decoy oligonucleotides effectively competed with the Oct-1 consensus probe for an Oct-1 binding *in vitro*

**Figure 1:**
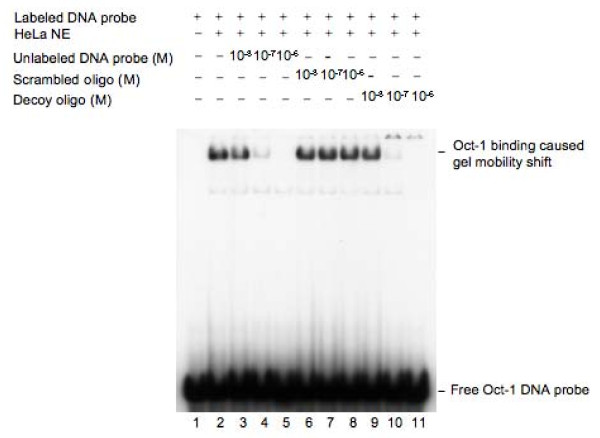
**Determination of the competitive binding of the Oct-1 decoy oligonucleotide *in vitro***. The radioactively labeled Oct-1 consensus DNA probe was incubated with HeLa nuclear extracts at room temperature for one hour and then analyzed by polyacrylamide gel electrophoresis using a 6% gel. Some reactions also contained the unlabeled Oct-1 decoy oligonucleotide or the scrambled oligonucleotide for competitive binding. The Oct-1 binding-caused gel mobility shift was confirmed by a western blot hybridization assay using an Oct-1 antibody (data not shown).

We first tested whether the decoy oligonucleotide designed for the Oct-1 transcription factor can effectively compete with the Oct-1 consensus probe *in vitro*. For this study, a 24 bp DNA fragment containing the Oct-1 consensus sequence was radioactively labeled with [γ-^32^P]-ATP and used as a probe for the *in vitro *DNA-protein binding study. The DNA probe was incubated with HeLa nuclear extracts at room temperature for one hour to allow for the Oct-1 transcription factor to bind to the DNA probe. For a competitive binding, the HeLa nuclear extracts were incubated with both the radioactively labeled Oct-1 DNA probe and the unlabeled Oct-1 decoy oligonucleotide at room temperature for one hour. The reactants were then analyzed by polyacrylamide gel electrophoresis using a 6% gel (Fig. 1). The binding of the Oct-1 transcription factor caused a mobility shift of the Oct-1 DNA probe in the gel (Fig. 1, lane 2 vs lane 1). When the HeLa nuclear extracts were incubated with both the radioactively labeled Oct-1 DNA probe and the unlabeled Oct-1 decoy oligonucleotide, however, a dose-dependent reduction in the level of the shifted Oct-1 DNA probe was observed (Fig. 1, lanes 9–11). The Oct-1 transcription factor binding-caused gel mobility shift of the Oct-1 DNA probe was completely diminished when 1 × 10^-6 ^M of the Oct-1 decoy oligonucleotide was supplemented in the binding reaction (Fig. 1, lane 11 vs lane 2). As a control, no clear reduction in the level of the shifted Oct-1 DNA probe was observed when similar concentrations of the scrambled oligonucleotide were supplemented into the binding reactions (Fig. 1, lanes 6–8). This result suggests that the Oct-1 decoy oligonucleotide effectively competes with the Oct-1 consensus DNA probe for the binding of the Oct-1 transcription factor.

### The Oct-1 decoy oligonucleotide treatment did not affect cell viability

**Figure 2:**
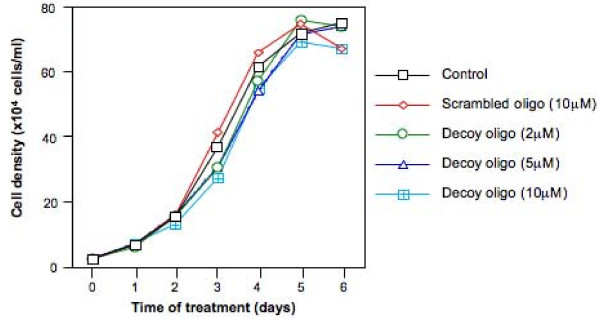
**The cell viability of K562 cells under the Oct-1 decoy oligonucleotide treatment**. The K562 cells were seeded at a density of 2 × 10^4 ^cells/ml and treated with either the scrambled oligonucleotide or the Oct-1 decoy oligonucleotide at the concentrations indicated. The cell density was determined at various time points (0, 1, 2, 3, 4, 5 and 6 days). The cell growth curves represent the mean data of three independent experiments.

To determine if the Oct-1 decoy oligonucleotide treatment could result in reduced cell viability, we studied the cell viability of the K562 human erythroleukemia cells under the Oct-1 decoy oligonucleotide treatment. The K562 cells were seeded at a density of 4 × 10^3 ^cells/ml and treated with the Oct-1 decoy oligonucleotide at various concentrations (0, 2, 5, and 10 *μ*M). As controls, some K562 cells were either untreated or treated with the scrambled oligonucleotide (10 *μ*M). The cell density was determined at various time points (1, 2, 3, 4, and 5 days) and the cell growth curve was determined for each treatment (Fig. 2). The cell growth was not significantly affected by the Oct-1 decoy oligonucleotide treatment even with the oligonucleotide at concentrations as high as 10 *μ*M (Fig. 2).

### The Oct-1 decoy oligonucleotide treatment caused an increase in expression of the *γ*-globin gene in K562 cells

The results obtained from our *in vitro *protein binding assays revealed a strong competitive binding of the Oct-1 decoy oligonucleotide for the Oct-1 transcription factor. To determine if the Oct-1 decoy oligonucleotide can induce expression of endogenous *γ*-globin genes, the K562 cells were treated with the Oct-1 decoy oligonucleotide and the effect of the decoy oligonucleotide treatment on *γ*-globin expression was determined.

**Figure 3:**
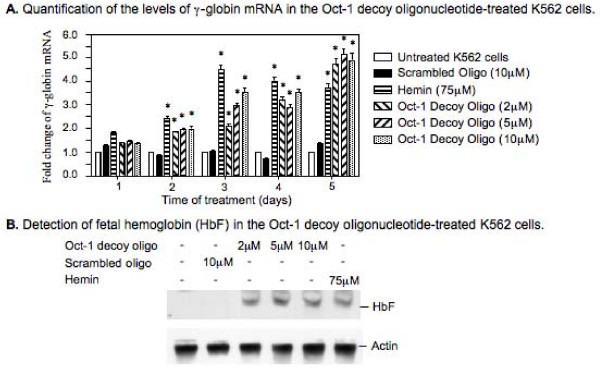
**The Oct-1 decoy oligonucleotide-induced expression of the endogenous *γ*-globin genes in K562 cells**. The K562 cells were treated with either the decoy or the scrambled oligonucleotides at indicated concentrations. As a positive control, some K562 cells were treated with 75 μM hemin. Total RNA was isolated from both untreated and treated cells at various time points following the treatment. The level of *γ*-globin mRNA was determined by a reverse transcription-based quantitative PCR (real time PCR). In order to determine the level of HbF in the treated K562 cells, the cells were treated with the decoy or the scrambled oligonucleotide for four days and then harvested and analyzed by western blots using an antibody that recognized the HbF. (A) The Oct-1 decoy oligonucleotide treatment-induced *γ*-globin transcription as determined by real time PCR assay. The level of the *γ*-globin mRNA in the untreated K562 cells was counted as 100% and the levels of the *γ*-globin mRNA in the treated K562 cells were calculated as relative levels to that of the untreated K562 cells. The results are from at least three independent experiments. (B) The Oct-1 decoy oligonucleotide treatment-induced accumulation of HbF in the K562 cells. The results are from three individual experiments. * Statistical significance between the untreated and the treated K562 cells at the same time point with p < 0.01.

We first determined the Oct-1 decoy oligonucleotide treatment-induced transcription of the *γ*-globin gene. The K562 cells were either untreated or treated with the Oct-1 decoy oligonucleotide at various concentrations (0, 2, 5, and 10 *μ*M). As controls, some K562 cells were treated with the scrambled oligonucleotide (10 *μ*M) or hemin (75 *μ*M), an effective hemoglobin inducer [30-32]. At various time points, the cells were harvested and total RNA was isolated. A real time PCR assay was then performed to determine the level of the *γ*-globin mRNA in each RNA sample (Fig. 3A). When treated with the scrambled oligonucleotide at 10 *μ*M, no significant increase in the level of *γ*-globin mRNA was observed in the K562 cells (Fig. 3A). When treated with the Oct-1 decoy oligonucleotide, however, significant increases in the level of the *γ*-globin mRNA were detected in the K562 cells even with the Oct-1 decoy oligonucleotide at concentrations as low as 2 *μ*M. For example, when the K562 cells were treated with 5 μ MOct-1 decoy oligonucleotide for two, three, four and five days, the level of the *γ*-globin mRNA increased 1.92, 2.93, 2.85, and 5.13 fold respectively. As a positive control, when the K562 cells were treated with 75 *μ*M hemin for two, three, four, and five days, the levels of the *γ*-globin mRNA increased 2.41, 4.47, 4.00, and 3.71 fold respectively (Fig. 3A). This result suggests that the Oct-1 decoy oligonucleotide treatment increases the transcription of the *γ*-globin genes in the K562 cells as effectively as that of hemin, a well-known γ-globin inducer.

Although the results obtained from our real time PCR study demonstrated increases in transcription of the *γ*-globin genes in the Oct-1 decoy oligonucleotide-treated K562 cells, whether these increases in transcription led to increases in the protein level of fetal hemoglobin (HbF, α2γ2) was unknown. Therefore, we further performed an immuno-blotting study to determine the level of HbF in the Oct-1 decoy oligonucleotide-treated K562 cells (Fig. 3B). Indeed, the results obtained from our immuno-blotting studies revealed that the treatment of the Oct-1 decoy oligonucleotide (2, 5, or 10 *μ*M) resulted in increases in the level of HbF in the K562 cells (Fig. 3B). As a negative control, the treatment of the K562 cells with 10 *μ*M scrambled oligonucleotide did not cause a significant increase in the level of HbF (Fig. 3B). As a positive control, the treatment with 75 *μ*M hemin also resulted in an increase in the level of HbF in the K562 cells (Fig. 3B).

These results suggest that the treatment of K562 human erythroleukemia cells with the Oct-1 decoy oligonucleotide caused an increased expression of the endogenous *γ*-globin genes in the K562 cells.

### The Oct-1 decoy oligonucleotide treatment caused a reduction in binding of the Oct-1 transcription factor to the promoter region sequence of the endogenous *γ*-globin gene

**Figure 4:**
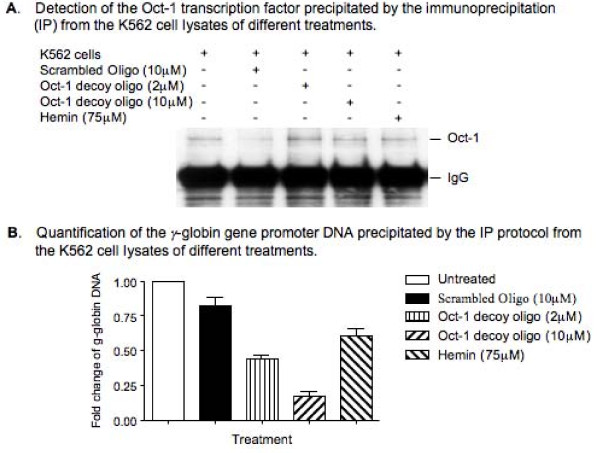
**The effect of Oct-1 decoy oligonucleotide treatment on binding of Oct-1 transcription factor to the endogenous γ-globin gene promoter region DNA in K562 cells**. The K562 cells were treated with either the decoy or the scrambled oligonucleotides for four days. The cells were fixed in 1% formaldehyde and sonicated to lyse the cells. An immunoprecipitation protocol was performed to pull down the Oct-1 transcription factor using an Oct-1 antibody and the Protein A-conjugated agarose beads. Half of the beads were analyzed by western blots to determine the level of Oct-1 transcription factor precipitated by the IP and the rest of the beads were treated in 5 M NaCl at 65°C for four hours to reverse the protein-DNA crosslinks and the recovered DNA was analyzed by a quantitative PCR (qPCR) protocol to determine the level of γ-globin gene promoter region DNA co-precipitated with the Oct-1 transcription factor. A pair of primers that bind to the *γ*-globin gene at the -350 to -330 and +50 to + 30 region sequences respectively was used in the qPCR study. (A) Detection of the Oct-1 transcription factor precipitated by the IP protocol. (B) Quantification of the levels of γ-globin gene promoter region DNA co-precipitated with the Oct-1 transcription factor in the IP assay. The results are from three independent experiments.

To determine whether the increased *γ*-globin gene transcription in the Oct-1 decoy oligonucleotide-treated K562 cells was caused by the competitive binding of the Oct-1 decoy oligonucleotide to the Oct-1 transcription factor, we further performed an immunoprecipitation (IP) study [33,34] to determine the binding status of the Oct-1 binding sites within the promoter region of the endogenous γ-globin gene under the Oct-1 decoy oligonucleotide treatment. Both untreated and the Oct-1 decoy oligonucleotide-treated K562 cells were fixed in 1% formaldehyde and sonicated to shear the chromosomal DNA into small fragments. The Oct-1 transcription factor was then immunoprecipitated from both the untreated and the decoy oligonucleotide-treated K562 cells by the IP protocol using an Oct-1 antibody and Protein A-conjugated agarose beads. As controls, the Oct-1 transcription factor was also immunoprecipitated from the scrambled oligonucleotide and hemin-treated K562 cells. Half of the beads were analyzed by western blot to determine the level of Oct-1 protein precipitated from each cell lysate (Fig. 4A) and rest of the beads were analyzed by a quantitative PCR assay to determine the level of the *γ*-globin gene promoter region DNA co-precipitated with the Oct-1 transcription factor from each cell lysate (Fig. 4B). The results of our western blots revealed that similar amounts of Oct-1 transcription factor were precipitated from individual cell lysates, suggesting a very successful IP of the study. The results of our quantitative PCR studies indicated that the Oct-1 decoy oligonucleotide treatment caused significant decreases in the level of the endogenous *γ*-globin gene promoter region DNA co-precipitated with the Oct-1 transcription factor by the IP protocol. In comparison to the amount of *γ*-globin gene promoter region DNA co-precipitated with the Oct-1 transcription factor in the untreated K562 cells, only 44% and 17% of the *γ*-globin gene promoter region DNA was co-precipitated with the Oct-1 transcription factor when the K562 cells were treated with 2 *μ*M and 10 *μ*M Oct-1 decoy oligonucleotide, respectively. When the K562 cells were treated with 10 μM scrambled oligonucleotide, however, more than 83% of the *γ*-globin gene promoter region DNA was still co-precipitated with the Oct-1 transcription factor. The treatment of K562 cells with 75 *μ*M hemin also resulted in a decrease in the level of the co-precipitated *γ*-globin gene promoter region DNA to 61% of the untreated K562 cells. This result revealed that the treatment of K562 cells with the Oct-1 decoy oligonucleotide caused a dose-dependent decrease in the binding of Oct-1 transcription factor to the endogenous *γ*-globin gene promoter region DNA sequence, suggesting that the Oct-1 decoy oligonucleotide effectively competed with the endogenous *γ*-globin gene promoter region Oct-1 consensus sequences for binding with the Oct-1 transcription factor, which resulted in activation of the endogenous *γ*-globin gene and increases in the level of *γ*-globin mRNA and HbF in the K562 cells.

## Discussion

In this work, we have studied the possibility of using a decoy oligonucleotide targeting the Oct-1 transcription factor to induce expression of the endogenous *γ*-globin gene. The results obtained from our *in vitro *protein binding study indicate that the Oct-1 decoy oligonucleotide effectively competes with the Oct-1 consensus DNA probe for binding to the Oct-1 transcription factor. The results obtained from our Oct-1 decoy oligonucleotide treatment studies demonstrate that the treatment of the K562 cells with the Oct-1 decoy oligonucleotide results in an increase in transcriptions of the *γ*-globin genes and an increase in accumulation of the HbF in the K562 cells. These results provide strong evidence to suggest that the decoy oligonucleotide targeting the Oct-1 transcription factor consensus sequence can be used to induce expression of the endogenous *γ*-globin gene. Since increased expression of the *γ*-globin gene has already shown its clinical benefit in the treatment of β-globin disorders such as sickle cell anemia and β-thalassemia, this work provides a novel approach for the treatment of these diseases. In addition, the knowledge obtained from this study may also lead to innovative strategies for the treatment of many other disease conditions via manipulating expression of the desired target genes through the decoy oligonucleotides.

The target of the decoy oligonucleotide used in this study is the Oct-1 transcription factor consensus sequence. It is known that the endogenous *γ*-globin genes promoter region contains three Oct-1 transcription factor-binding sites, the -280, -220 and -175 binding sites, respectively [1]. The naturally occurring mutations at the -175 Oct-1 binding-site of the *γ*-globin gene lead to elevated levels of HbF in individuals with hereditary persistence of fetal hemoglobin (HPFH) [11-13]. Biochemistry studies also reveal that these mutations diminish the binding of the Oct-1 transcription factor to the site. The results obtained from our previous studies demonstrated that mutations generated at the -280 Oct-1 binding site of the *γ*-globin gene cause increased expressions of the *γ*-globin gene [14]. The results obtained from this study further reveal that the treatment of the K562 cells with the Oct-1 decoy oligonucleotide leads to the activation of the endogenous *γ*-globin genes. All of these results suggest that the Oct-1 transcription factor negatively regulates the transcription of the endogenous *γ*-globin gene expression. Therefore, these results provide important insight into the mechanism of *γ*-globin gene regulation and a possible mechanism regarding the *γ*-globin gene silencing.

Many studies have demonstrated the down-regulation of the target genes' expression using decoy oligonucleotides [15,16,20-29,35-38]. In comparison, much less work has been conducted in the exploration of up-regulation of gene expression for the desired target genes using the decoy oligonucleotide strategy although the clinical relevance of up-regulation of target genes for the treatment of many disease conditions clearly exists. One of the obstacles for this up-regulation is to identify the transcription factors that negatively regulate transcription of the target genes. It is also a great challenge to develop a methodology that can sequence-specifically disrupt the interactions between the transcription factors and their consensus sequences. The work described here takes advantage of the well-studied human *γ*-globin gene by disrupting the binding of Oct-1 transcription factor to the endogenous *γ*-globin gene promoter region Oct-1 consensus sequences using a decoy oligonucleotide, and therefore, achieving activation of the endogenous *γ*-globin genes in the treated cells. A similar strategy can also be applied to other target genes by targeting different transcription factor consensus sequences. Therefore, the work described in this study has broad implications in the treatment of many disease conditions.

The results obtained from our K562 cell study revealed that the transcription of the γ-globin genes was significantly increased when the K562 cells were treated with the Oct-1 decoy oligonucleotide at concentrations as low as 2 *μ*M, which is the lowest concentration of the decoy oligonucleotide used in the study. Therefore, it is likely that the transcription of the *γ*-globin genes can be achieved at even lower concentrations of the decoy oligonucleotide. This result suggests that the concentration of the Oct-1 decoy oligonucleotide required to induce transcription of the *γ*-globin genes is much lower than the concentrations of antisense oligonucleotides used in most studies [39-42]. One possible explanation for this difference is the target choice in the strategies: when the antisense strategy is used to regulate expression of target genes, high levels of antisense oligonucleotides need to be maintained inside the cells in order to effectively inhibit translation from the mRNA, which is continuously transcribed throughout the treatment; when the decoy oligonucleotide strategy is used, however, the limited copy numbers of the target genes result in the requirement of much fewer molecules of decoy oligonucleotide for efficient regulation of transcription of the target genes. Therefore, the decoy oligonucleotides strategy may provide a better approach in regulating expression of the target genes than that of the antisense strategy.

The results obtained from our real time PCR analysis indicated that the transcription of the *γ*-globin genes was maintained at high levels for a relatively long period of time following a single Oct-1 decoy oligonucleotide treatment. The mechanism that leads to this long duration of transcription is unknown. One possibility is that the double-stranded phosphorothioate-modified decoy oligonucleotide is more stable than that of the single-stranded phosphorothioate-modified oligonucleotides inside cells and the requirement of fewer molecules of the decoy oligonucleotide leads to this relatively long period of transcription for the *γ*-globin gene. It is also possible that the disruption of the Oct-1 transcription factor binding to its endogenous gene targets due to the decoy oligonucleotide has a prolonged effect on transcription of the genes, which can also lead to the long duration of transcription of the *γ*-globin gene following the Oct-1 decoy oligonucleotide treatment. Further studies are needed in order to determine the mechanism that causes this extended transcription duration of the *γ*-globin gene following the Oct-1 decoy oligonucleotide treatment.

The results obtained from our IP studies revealed that the Oct-1 decoy oligonucleotide treatment led to reduced Oct-1 transcription factor binding at the *γ*-globin promoter. This provides strong evidence to suggest that the Oct-1 decoy oligonucleotide induces expression of the *γ*-globin genes through its competitive binding with the Oct-1 transcription factor, resulting in activation of the *γ*-globin genes. The hemin treatment also led to reduced Oct-1 binding at the *γ*-globin promoter. Although the mechanism underlying this effect is not known, it is possible that the induced transcription of the *γ*-globin genes by the hemin reduces the binding of the Oct-1 to the *γ*-globin promoter.

The decoy oligonucleotides used in this study are phosphorothioate-modified oligonucleotides. The results obtained from our cell viability study indicate that the cells are relatively tolerant towards the decoy oligonucleotide treatment even with the decoy oligonucleotide at concentrations as high as 10 *μ*M. The *in vitro *protein-DNA binding results also suggest that the Oct-1 transcription factor has a strong binding affinity towards the decoy oligonucleotide. The gene expression studies also demonstrate the cell permeability for the decoy oligonucleotide. Therefore, this modified decoy oligonucleotide is effective in inducing expression of the endogenous *γ*-globin genes. However, other modifications (*e.g*. peptide nucleic acids (PNA)) are also available in the oligonucleotide synthesis. Although some studies have already used these modifications in the decoy oligonucleotide synthesis [15,27,37], whether or not these modifications can be used to induce expression of endogenous gene targets is still largely unclear. Further studies are needed to evaluate the effect of these modifications on decoy oligonucleotide-induced endogenous gene expression.

## Competing interests

The authors declare that they have no competing interests.

## Authors' contributions

XSX is involved in the overall study design, performed data analysis, and drafted the manuscript. XH carried out the gel mobility study, cell treatment, and real time PCR study. GW is involved in the overall study design, carried out the chromatin-immunoprecipitation (ChIP) experiments, and involved in the manuscript preparation.
